# Surface Display of *porcine circovirus* type 2 antigen protein cap on the spores of *bacillus subtilis 168*: An effective mucosal vaccine candidate

**DOI:** 10.3389/fimmu.2022.1007202

**Published:** 2022-09-15

**Authors:** Weijie Li, Jianzhen Li, Xixi Dai, Minggang Liu, Abdul Khalique, Zhenghua Wang, Yan Zeng, Dongmei Zhang, Xueqin Ni, Dong Zeng, Bo Jing, Kangcheng Pan

**Affiliations:** ^1^ Animal Microecology Institute, Department of Animal and Plant Quarantine, College of Veterinary Medicine, Sichuan Agricultural University, Chengdu, China; ^2^ College of Animal Husbandry and Veterinary, Chengdu Agricultural College, Chengdu, China; ^3^ College of Animal Science and Technology, Chongqing Three Gorges Vocational College, Chongqing, China

**Keywords:** *Porcine circovirus type 2*(PCV2), capsid protein, *Bacillus subtilis*, spore surface display, oral mucosal vaccine, mucosal immunity

## Abstract

The oral mucosal vaccine has great potential in preventing a series of diseases caused by porcine circovirus type 2 (*PCV2*) infection. This study constructed a recombinant Bacillus subtilis RB with *PCV2* Capsid protein (*Cap*) on its spore surface and *cotB* as a fusion partner. The immune properties of the recombinant strain were evaluated in a mouse model. IgA in intestinal contents and IgG in serum were detected by enzyme-linked immunosorbent assay (ELISA). The results demonstrated that recombinant spores could activate strong specific mucosal and humoral immune responses. In addition, spores showed good mucosal immune adjuvant function, promoting the proliferation of CD3+, CD4+ and CD8+ T cells and other immune cells. We also found that the relative expression of inflammatory cytokines such as IL-1β, IL-6, IL-10, TNF-α and IFN in the small intestinal mucosa was significantly up-regulated under the stimulation of recombinant bacteriophage. These effects are important for the balance of Th1/Th2-like responses. In summary, our results suggest that recombinant *B. subtilis* RB as a feed additive provides a new strategy for the development of novel and safe *PCV2* mucosal subunit vaccines.

## Introduction

*Porcine circovirus* (*PCV*) is a member of the genus *Circovirus* of the *Circoviridae* family. The genotypes mainly include *PCV1*, *PCV2*, *PCV3* and *PCV4* (1). *PCV2* and *PCV3* are pathogenic, and *PCV2* is the main epidemic genotype. In addition, *PCV2* is one of the smallest known animal viruses (about 17nm), with an icosahedral structure and single-stranded negative DNA genome without a viral envelope. The full-length DNA of *PCV2* is about 1700 bp, and its major open reading frames (ORFs) are orf1 and orf2 (2). Orf1 encodes two replication-related proteins (rep and rep’), and orf2 encodes viral capsid protein-*Cap*. Orf2 sequence is the main reference for *PCV2* typing (3, 4). *Cap* protein is the main antigen protein that induces the production of *PCV2-*specific neutralizing antibodies. It has highly conserved epitopes and the ability to induce strong immune responses. At present, *Cap* protein is commonly used in the development of subunit vaccines and the detection of *PCV2 (* 5). *PCV2* can be divided into various genotypes (*PCV2*a-*PCV2*i). Since 2014, *PCV2*d has replaced *PCV2*b as the most common strain in China (6). Commercial vaccines have cross-protection against many genotypes, but it is more targeted to use the *PCV2* gene as a vaccine candidate in the current *PCV2* epidemic (7). *PCV2* infection is usually accompanied by a decrease in lymphocytes or monocytes, which further leads to immunosuppression of the host (8). *PCV2* often causes Post-weaning Multisystemic Wasting Syndrome (PMWS), Porcine Dermatitis and Nephropathy Syndrome (PDNS), Congenital Tremor, Proliferative and Necrotizing Pneumonia (PNP), Porcine Respiratory Disease Complex (PRDC), reproductive disorders (late abortion) and other diseases, with high morbidity and mortality (9). In the clinic, *PCV2* often forms multiple infections with Porcine Reproductive and Respiratory Syndrome Virus (PRRSV), Classical Swine Fever Virus (CSFV), Porcine Parvovirus (PPV) and Porcine Pseudorabies Virus (PRV). In addition, porcine circovirus often forms secondary co-infection with *Haemophilus parasuis*, *Streptococcus* and *Actinobacillus pleuropneumoniae*, which brings massive economic losses to the pig industry (10).

Currently, there is no effective treatment for *PCV2* infection. The use of vaccines is an important strategy to prevent and treat *PCV2* infection. The main commercial *PCV2* vaccines in clinical use at this stage are whole virus inactivated vaccines and subunit vaccines, which have played an important role in the prevention and control of *PCV2*. Vaccination can reduce the damaging effect of *PCV2* on porcine lymphoid tissues, reduce viremia, and significantly decrease the replication level of *PCV2* in pigs. Vaccination with different commercial inactivated vaccines reduced the mortality of PCV2-infected piglets by an average of 72% (11–13). Inactivated vaccines are widely used because of their safety, ease of production, genetic stability. A large number of studies have confirmed that the initial infection of *PCV2* mainly occurs on the mucosal surface, especially in the respiratory tract and gastrointestinal tract (14–16). *PCV2* first reaches the lung and small intestinal mucosa after infection *via* the nasal and gastrointestinal routes. In the intestine it attacks Peyer’s patches, induces accelerated apoptosis of lymphocytes and causes immunosuppression. In addition, a large amount of intestinal mucosa is shed and necrosis occurs, and the intestinal mucosa loses its immune barrier function, which is a reason why *PCV2* is prone to co-infection with other pathogens. However, most licensed vaccines administered by the parenteral route do not induce protective mucosal immunity. This may only protect clinical diseases but cannot eliminate infection at local mucosal invasion sites (17). And most commercial vaccines do not induce both humoral and cellular immunity in the body. Thus, it is necessary to develop a more effective mucosal vaccine against *PCV2* infection in pigs.

*Bacillus subtilis* (*B. subtilis*) is an aerobic, spore-forming Gram-positive bacterium. It has strong secretion ability, simple cell wall structure, clear genetic background and non-toxicity. In addition, it is a non-pathogenic and non-invasive bacterium. It is GRAS (generally recognized as safe) and is widely used as a probiotic and food additive in humans and other animals. In 2001, Isticato (18) successfully displayed the 459 amino acid residues at the C-terminal of tetanus toxin protein on the spore surface of *B. subtilis* for the first time and induced a specific immune response. At present, the surface display technology of B. subtilis has been successfully applied to the immunization of *Porcine Epidemic Diarrhea Virus* (19), *Porcine Rotavirus* (20), *Clostridium Difficile* (21), and *Clonorchis Sinensis* (22).

Mucosal vaccines have more routes of immunity than traditional injectable vaccines, including oral, eye-drop and intranasal immunization. It is easy to operate, has low cost and has little stimulation to the host in clinical applications. In addition, oral vaccines based on spore surface display technology can deliver antigens through the mucosal route and induce systemic and mucosal immune responses. Spores of *B. subtilis* can regulate the balance of Th1 and Th2 immune responses, and spores have good immunoadjuvant function (23, 24). These studies suggest that natural, non-pathogenic and non-symbiotic spores of *B. subtilis* induce and enhance immune responses, and have great potential for future clinical applications.

In this study, the recombinant *B. subtilis* RB which displayed *PCV2 Cap* protein on the surface of spores was successfully constructed. The fusion gene *cotB-tCap* was integrated into the genome of *B. subtilis* 168 using the integrative plasmid pDG364. In addition, we evaluated the specific immune response induced by *B. subtilis* RB in a mouse model and further analyzed the immune adjuvant effect of the recombinant spores. The above work provides a new idea for the prevention of *PCV2*-related diseases in the future.

## Materials and methods

### Plasmids, bacteria, strains, cell lines, and primer sequences

The bacteria, virus strains, cell lines and plasmids used in this study are listed in **Supplementary Table S1**.

### Cloning of truncated *Cap* gene and typing of virus

The diseased tissues of pigs infected with *PCV2* were collected by the Pig Farm Health Testing and Evaluation Center of Sichuan Agricultural University, and viral DNA was extracted using DNeasy Blood & Tissue Kit (QIAGEN, Germany) according to the instructions. We named this strain *PCV2* SC2020. We designed primers *tCap*-F1 and *tCap*-R1 to amplify the truncated *tCap* gene according to the research by Chen (25). The PCR product was purified and cloned into pUCm-T using T Vector Rapid Cloning Kit (Sangon Biotech, Shanghai, China). After sequencing the recombinant plasmid pUCm-T-*tCap*, implemented point mutation to the codons to avoid restriction sites that would be used (commissioned Tsingke Biotechnology Co., Ltd.). The sequences were compared with other representative strains of *PCV2* genotype and a phylogenetic tree was constructed using MEGA 6.0 software. Other representative *PCV2* strains used for comparison are shown in **Supplementary Table S2**. LB agar medium with 0.1 mg/mL ampicillin was used for screening.

### Construction of pET-32a-t*Cap* and prokaryotic expression of t*Cap*

The mouse derived anti-*PCV2* hyperimmune serum was prepared according to the method of Li (20) and their antibody titers were assayed. Specifically, 5 BALB/c mice were intraperitoneally injected with 100 μL of commercial vaccine (Inactivated *PCV2* Vaccine<Strain WH>, Zhongmu, Chengdu, China). The immunization was boosted a week later and the mouse serum was collected after 4 immunizations. The serum antibody titer was detected by indirect ELISA (26) (Antigen-coated plates encapsulated with PCV2 Cap protein were purchased from JNT, Beijing, China.). The collected mouse sera were diluted to 1:100 using PBST and then subjected to two-fold serial dilution, and the maximum dilution for positive reactions was the serum antibody potency.

The pUCm-T-*tCap* and pET-32a empty plasmids were subjected to double digestion reaction using *BamH* I and *EcoR* I (Takara Bio, Dalian, China). The target fragments were ligated to obtain the expression plasmid pET-32a-*tCap* and then transformed into *E. coli* BL21.The expression of recombinant protein was induced by isopropyl-β-D-thiogalactoside (IPTG) at a final concentration of 1 mM. Collected 1 mL of bacterial liquid at 1 h intervals, and the collection was continued for 7 h. The pET-32a empty plasmid was set as a blank control. The collected bacterial liquid was sonicated and centrifuged at 10,000 g for 1 min to take the supernatant. The supernatant was subjected to 12% SDS-PAGE and transferred to nitrocellulose membranes (Solarbio, Beijing, China). Incubated overnight at 4°C with mouse-derived *PCV2* hyperimmune serum (Diluted to 1:100 in PBST) as the primary antibody. Wash with TBST and then nitrocellulose membrane was incubated with HRP-conjugated goat anti-mouse IgG at 37°C for 1.5 h. After washing with TBST, the blot filters were visualized using DAB substrate.

### Construction and transformation of recombinant integration plasmid

Used *tCap*-F2/*tCap*-R2 primers to replace the restriction site of *tCap.* Based on our previous work (19), the recombinant plasmid pDG364-*cotB-tCap* was constructed. The method was the same as pET-32a-*tCap* in 2.3. Competent cells of *B. subtilis* 168 were prepared according to the method of Julkowska (27). The linearized plasmid has higher homologous recombination efficiency after transforming into cells (28), so we linearized pDG364-*cotB*-*tCap* with restriction endonuclease *Xba* I. Added an appropriate amount of linearized pDG364-*cotB*-*tCap* into 500 μL of competent cells, mixed gently and shaked slowly at 37°C (80 r/min) for 1 h. The integrated plasmid pDG364-*cotB*-*tCap* uses the upstream and downstream of the amylase gene of *B. subtilis* as its homology arm. When the plasmid pDG364-*cotB*-*tCap* was transformed into *B. subtilis* 168, under the pressure of antibiotics, its homology arm was exchanged with the chromosome of *B. subtilis* 168 by homologous double-crossover recombination. The selection medium was LB medium containing 5μg/mL of chloramphenicol. used 1% starch nutrient agar medium to screen the positive strains with amylase gene deletion. Extracted the genome of recombinant bacteria and used several different pairs of primers **Supplementary Table S1** for PCR identification.

### Preparation of spores and indirect immunofluorescence

Used Difco Sporulation Medium (DSM) to sporulate the *B. subtilis* RB and *B. subtilis* 168 according to the nutrient depletion method (29). The purified spores were fixed and observed by immunofluorescence microscopy to identify whether the *tCap* protein was successfully displayed on the spore surface. The primary antibody was mouse-derived anti-*PCV2* hyperimmune serum (Diluted to 1:100 in PBST), and the secondary antibody was fluorescein Cy3- conjugated goat anti-mouse IgG (Diluted to 1:100 in PBST, Biomed, Beijing, China). Also, serum from unimmunized mice was used as a primary antibody, then indirect immunofluorescence of *B. subtilis* RB spores were performed with Cy3-conjugated goat anti-mouse IgG. Excited with a 532nm laser (green light), Cy3 will fluoresce orange.

### Grouping and immunization of experimental animals

A hundred of female BALB/c mice (2-week-old) were pre-fed for 7 days. The mice were divided into 5 groups, with 4 replicates in each group and 5 mice in each replicate (**Table 1**). There were no significant differences in the body weight of mice between groups. *B. subtilis* 168 and *B. subtilis* RB were fermented and sporulated using DSM, and the number of spores was adjusted to immunize mice according to the immunization program in **Table 1**. The nutrition of the mouse diet fully meets the growing needs of the mice, and the number of spores added referred to Tang and Dai (19, 30). Each of the mice were fed 3-5 g per day.

**Table 1:** Program for the vaccination experiment.

Group	Strains for immunization	Method ofAdministration	Dose	Time ofAdministration
Ctrl	Blank control	Blank control	Blank control	Blank control
168-spores/mixed feeding	*B. subtilis* 168	Fed the mixed diet	2.5×10^6^ CFU/g feed	The whole period
RB-spores/gavage	*B. subtilis* RB	Gavage	2.0×10^10^CFU/mL*	Days 1-3, 14-16, and 28-30
RB-spores/mixed feeding	*B. subtilis* RB	Fed the mixed diet	2.5×10^6^ CFU/g feed	The whole period
Inactivated vaccine	Commercial vaccine	Intraperitoneal injection	100μL/per mouse	Days 1, 14, and 28

* The bacterial concentration was adjusted to 2.0×10^10^CFU/mL with normal saline. On day 1-3, 14-16, and 28-30, each mouse was given 0.1 mL intragastric administration every day for 3 consecutive days.

All animal experiments were performed in accordance with the guidelines for the care and use of laboratory animals and approved by the Institutional Animal Care and Use Committee of Sichuan Agricultural University (approval number: DY2020203062).

### Collection of samples

Serum and small intestine contents were collected on days 0, 14, 28, and 42. Euthanized 5 mice of each group at the set time for sampling. The small intestine tissue which the contents were removed was placed in a centrifuge tube and stored at -80°C.

The indirect ELISA method described by Liu (31) was used to detect the serum IgG (Immunoglobulin G)and sIgA (Secretory immunoglobulin A)in the small intestine, antigen-coated plates encapsulated with PCV2 Cap protein were purchased from JNT, Beijing, China. The serum and contents of the small intestine were diluted to 1:100 with PBST, and the unimmunized mouse serum and hyperimmune serum were set as negative and positive control. The secondary antibodies were HRP-conjugated goat anti-mouse IgG (Diluted to 1:4000 in PBST, Biomed, Beijing, China) and HRP-conjugated goat anti-mouse IgA (Diluted to 1:10000 in PBST, Acbam, U. K). The levels of sIgA in the small intestinal contents and IgG in serum were expressed as *P/N* values:

*P/N*= OD450 of sample well/OD450 of negative control well

### Isolation and culture of the virus

The spleen tissue of pigs infected with *PCV2* was homogenized by adding appropriate amount of PBS, freeze-thawed three times, and then added penicillin-streptomycin incubated for 24h at 4°C. Centrifugated to take the supernatant, then used 0.22μm microporous filter sterilized and obtained the virus stock solution. PK15 cells were cultured in DMEM medium with 10% FBS until the confluence rate was 50%. Discarded the medium and added 1 mL of virus stock solution and incubated at 37°C for 2 h. D-glucosamine could significantly promote the proliferation of *PCV2* in PK15 cells (32). After discarding the virus solution, cells were treated with 300 mmol/L D-glucosamine at 37°C for 30 min. Then continued the culture with DMEM medium containing 2% FBS for 72 h. Collected virus by repeated freezing and thawing 3 times.

After blind passage for 3 generations, the TCID50 of *PCV2* SC2020 was determined according to the IPMA method (33). Briefly, the *PCV2* virus solution was serially diluted with serum-free DMEM medium from 10^-1^ to 10^-8^, and 100 μL of the virus solution and PK15 cells suspension were inoculated into a 96-well plate. Until the confluence rate was 80%, treated with 300 mmol/L D-glucosamine at 37° C for 30 min. Then continued the culture with DMEM medium containing 2% FBS for 72 h. Fixed with 90% acetone fixative and blocking with 5% BSA for 2 h at room temperature. Mouse-derived *PCV2* hyperimmune serum (Diluted to 1:100 in PBST) was added and incubated at 4°C overnight. After washing, add HRP-conjugated goat anti-mouse IgG (diluted to 1:4000 in PBST) and incubate at 37°C for 1 h, then used DAB substrate for color development, observed the brown-yellow signal under the microscope and calculated the TCID50 according to the Reed-Muench method.

### Microneutralization test

To determine whether the serum antibodies induced by recombinant spores could effectively neutralize the virus, we constructed an *in vitro* infection model for virus neutralization test.PK15 cells were cultured in a 96-well cell culture plate, when the confluence rate reached about 50%, performed microneutralization tests according to the method of Chen (25). Briefly, the serum samples (50 μL) collected on 42nd day were heat inactivated at 56°C for 30 min and two-fold serial diluted from 2^-1^ to 2^-8^ in DMEM medium. 50 μL of *PCV2* SC2020 (200 TCID50) were mixed with equal volume of serum and co-incubated at 37°C for 1 h. The mixed solution (Diluted serum and *PCV2* virus) was then inoculated into a 96-well plate containing 40–50% confluent PK-15 cells, and each dilution of mixed solution was inoculated into 3 replicate wells. After incubating 72 hours at 37°C and 5% CO_2,_ the cells were washed twice with PBS and fixed with cold acetone/methanol (1: 1, V: V) at − 20°C for 20 min. Then blocked with 3% BSA in PBST for 1 h at room temperature. Incubated primary antibody using the same method as 2.8. Then incubated with Cy3-conjugated goat anti-mouse IgG and visualized by fluorescence microscopy. Titers were determined as the reciprocal of the final serum dilution with a 70% or greater reduction in fluorescence under fluorescence microscopy. Finally, the serum neutralizing antibody titers were calculated by the Reed-Muench method (25).

### Quantitative Real-time PCR detection of cytokine expression in small intestinal

Total RNA was extracted from small intestine samples, using HI Script^®^ III RT SuperMix (Vazyme, Nanjing, China) reverse transcribed RNA into cDNA, cDNA was diluted to an appropriate concentration and used as a Quantitative Real-time PCR (qPCR) reaction template.

Used qPCR to determine the relative expression abundance between IL-1β, IL-6, IL-10, IFN-γ, TNF-α and the internal reference gene β-actin in small intestine (34). The primer sequences used for qPCR are shown in **Supplementary Table S3**. The 2^-ΔΔCT^ method was used to calculate the relative expression of the target gene:

*ΔΔCT*= (CT, target gene - CT, β-actin) experimental group- (CT, target gene - CT, β-actin) control group.

### Detection of CD3+, CD4+, CD8+ T lymphocytes and IELs in small intestinal

The small intestine fixed in 4% paraformaldehyde were trimmed and dehydrated with alcohol gradient using a dehydrator (DIAPATH, Italy). After dehydration, the tissue soaked in wax was embedded in paraffin and serially sectioned with a thickness of 4 μm. Small intestinal tissue slices were deparaffinized in xylene and rehydrated in a decreasing concentration gradient of alcohol. The slices were placed in citrate antigen retrieval solution (PH 6.0, Servicebio, Wuhan, China) at 95°C for antigen retrieval, washed with PBS. Slices were blocked with 3% BSA at room temperature for 30 min. After removing the blocking solution, incubated with primary antibody at 4°C overnight. After washing with PBS, the corresponding HRP- conjugated secondary antibodies (Diluted to 1:4000 in PBS) were added, and incubated at room temperature for 1 h. Used DAB substrate to visualize and performed hematoxylin-eosin counterstaining.

Immunohistochemical slices images were scanned by an imaging system (Nikon, Japan), and the positive intensity was evaluated according to the method of Paschalis (35). Three slice samples were randomly selected from each group, and three regions were randomly selected from each slice sample for analysis (n=9). Histochemistry Score (H-score) is a histological scoring method for immunohistochemistry, which converts the number of positive cells and their staining intensity in each section into corresponding values to achieve the purpose of quantifying positive cells. First, the positive grades were scored: negative without coloration, 0 points; weak intensity with light yellow, 1 point; moderate intensity with brownish yellow, 2 points; strong intensity with tan, 3 points. Used Image-Pro Plus 6.0 software to calculate the weak, medium and strong positive areas in the measurement area, and the total area of positive tissues. H-score is a value between 0-300, the larger the value, the stronger the overall positive intensity.

*H-score*=∑(pi×i) = (percentage of weak intensity area×100) +(percentage of moderate intensity area×200) +(percentage of strong intensity area×300).

Referring to the method of Wang (36), randomly selected 5 slices of each tissue sample to image the target area using an Eclipse Ci-L photographic microscope (Nikon, Japan) (n=5). Then used the Image-Pro Plus 6.0 software analyze the images, and the standard unit was millimeters. Selected 100-fold field of view, five intact intestinal villi were selected from each intestinal tissue slice to measure the height and the number of IELs (Intestinal intraepithelial lymphocytes). Calculate the number of IELs per unit height:

*The number of IELs per unit height*= The number of IELs in this intestinal villi/Height of the intestinal villi

### Statistical analysis

For multiple group comparisons, one-way ANOVA followed by a Friedman test was applied. Data were statistically analyzed using IBM SPSS Statistics 27 (IBM Corporation, Armonk, New York, USA) and presented as mean ± standard deviation (SD). *P* value less than 0.05 was considered significant. Experimental data visualization was performed using Origin 2021b (OriginLab, Northampton, Massachusetts, USA).

## Results

### Cloning of truncated *Cap* gene and typing of the virus

The spleen of a *circovirus*-infected pig was collected from a pig farm in Sichuan province, China. The strain was named *PCV2* SC2020. The *tCap* gene was amplified and cloned into the pUCm-T vector. Agarose gel electrophoresis results showed that the length of *tCap* was 579 bp (**Supplementary Figure S1A**). The sequence was compared with other representative *PCV2* strains in NCBI, and the phylogenetic tree was constructed with MEGA 6.0 software (**Supplementary Figure S1B**). The results showed that the sequence had 100% homology with *PCV2* CH/HNZMD1/201406 (GenBank accession number: KX641112.1) and belonged to *PCV2*d. Sequencing results showed that the *tCap* gene contained restriction sites, and a sequencing company (Tsingke Biotechnology Co., Ltd.) was further commissioned to carry out point mutation of the codon of this gene (**Supplementary Table S4**).

### Construction of pET-32a-t*Cap* and prokaryotic expression of t*Cap*

**Figure 1:**
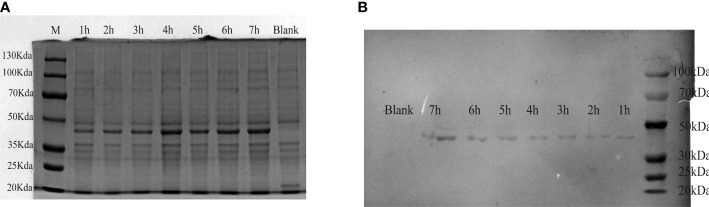
Analysis of expression products of *E. coli* BL21/pET-32a-*tCap*. **(A)** Visual analysis of protein by 12% SDS-PAGE. Line M, protein marker; line 1-7, expression product after adding IPTG for 1-7h; line 8: blank control. **(B)** Western blot analysis of transmembrane products.

The verified plasmid pET-32a-*tCap* was transformed into *Escherichia coli (E. coli)* BL21 and obtained a prokaryotic expression strain. *E. coli* BL21/pET-32a-*tCap* was induced by IPTG at 37°C. The expression product was analyzed by western blot and 12% SDS-PAGE. The protein bands were visualized by Coomassie brilliant blue (**Figure 1A**) and its molecular weight was about 48kDa, which was consistent with the theoretical value. In addition, the expression of the protein gradually increased with the extension of the induction time.

ELISA showed that the antibody potency of mouse-derived *PCV2* hyperimmune serum was 1:12800. Used it as primary antibody, and analyzed the expression product of *E. coli* BL21/pET-32a-*tCap* by western blot (**Figure 1B**). The results showed that protein staining bands appeared at 48 kDa. This indicated that the recombinant protein was successfully expressed and was responsive to the *PCV2* antibody.

### Construction and transformation of recombinant integration plasmid

**Figure 2:**
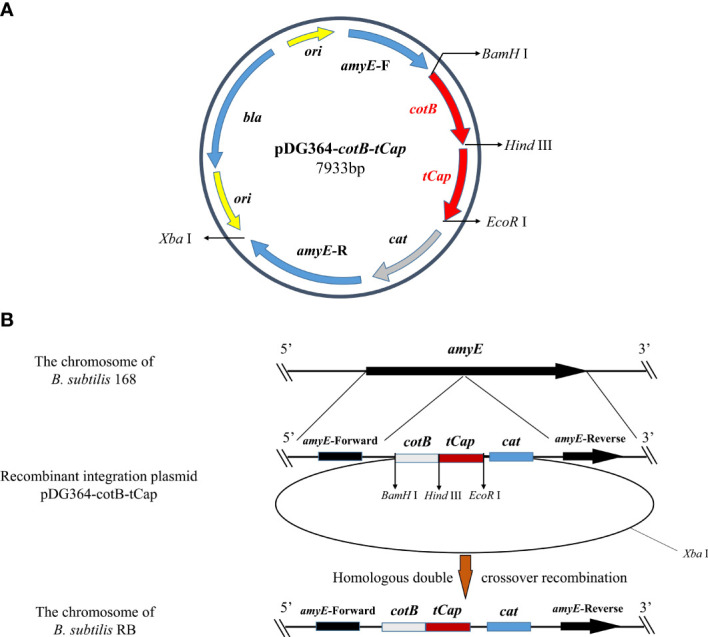
Homologous recombination between integration plasmids pDG364-*cotB*-*tCap* and the *B*. *subtilis* 168 genomes. **(A)** Map of the plasmid vector pDG364-*cotB*-*tCap2.* **(B)** Homologous double crossover recombination between the recombinant plasmid pDG364-*cotB*-*tCap* and the genome of *B*. *subtilis* 168.

The recombinant integration plasmid pDG364-*cotB*-*tCap* was verified by double restriction enzyme digestion (**Supplementary Figure S2A**) and then transformed it into *B. subtilis* 168 competent cells by chemical method. The integration plasmid pDG364 used upstream and downstream (**Figure 2A**) of the *B. subtilis* amylase gene as its homology arm. After the addition of antibiotics, the homologous arms were exchanged by homologous double cross recombination with the chromosomes of *B. subtilis* 168 (**Figure 2B**). The fusion gene *cotB*-*tCap* was inserted into the amylase gene of *B. subtilis* 168, and the amylase gene was destroyed. As shown in **Supplementary Figure S2B**, *B. subtilis* RB was grown on 1.5% starch medium and reacted with iodine solution. This indicated that the starch was not broken down and its amylase activity was lost. Primers *tCap* F2/R, *amyE* F/R, *amyE* F/*tCap* R2, and *tCap* F2/*amyE* R were used for PCR identification (**Supplementary Figure S2C**). Wild-type *B. subtilis* 168 was used as negative control. The product sizes were respectively 569 and 3557 bp (Line 1&2), none and about 1900 bp (Line 3&4), none and 1688 bp (Line 5&6), none and 594 bp (Line 7&8). Electrophoretic of PCR products showed that the size was consistent with the theoretical values, indicating that *B. subtilis* RB was successfully constructed.

### Preparation of spores and indirect immunofluorescence

**Figure 3:**
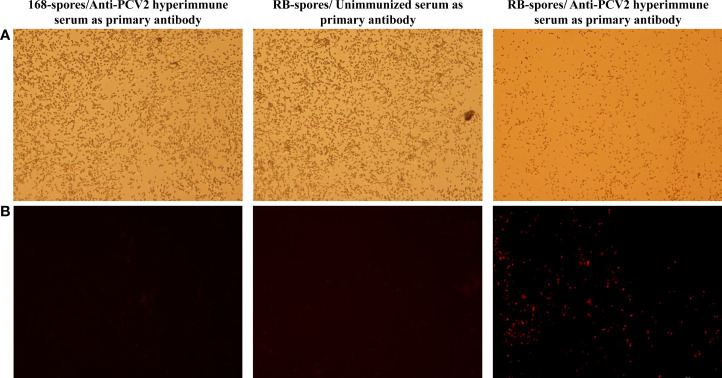
Indirect immunofluorescence assay of the sporulated *B*. *subtilis* RB and *B*. *subtilis* 168 strains. **(A)** Spores of *B*. *subtilis* 168 under and RB under 40×bright-field of fluorescence microscopy **(B)** Spores of *B*. *subtilis* 168 and RB under 40× fluorescence field, with different serum as primary antibodies and Cy3- conjugated goat anti-mouse IgG was used as secondary antibody. Only the spores of *B*. *subtilis* RB incubated with mouse-derived anti-*PCV2* hyperimmune serum emit fluorescent signals in the fluorescent field.

*PCV2* hyperimmune serum and Cy3-conjugated rabbit anti-mouse IgG were used respectively as primary antibody and secondary antibody for indirect immunofluorescence assay (**Figure 3**). The results showed that only the spores of *B. subtilis* RB emitted fluorescent signal. This indicated that the *tCap* protein was successfully displayed on the spore surface of *B. subtilis* RB.

### Detection of *PCV2*-specific antibodies by indirect ELISA

**Figure 4:**
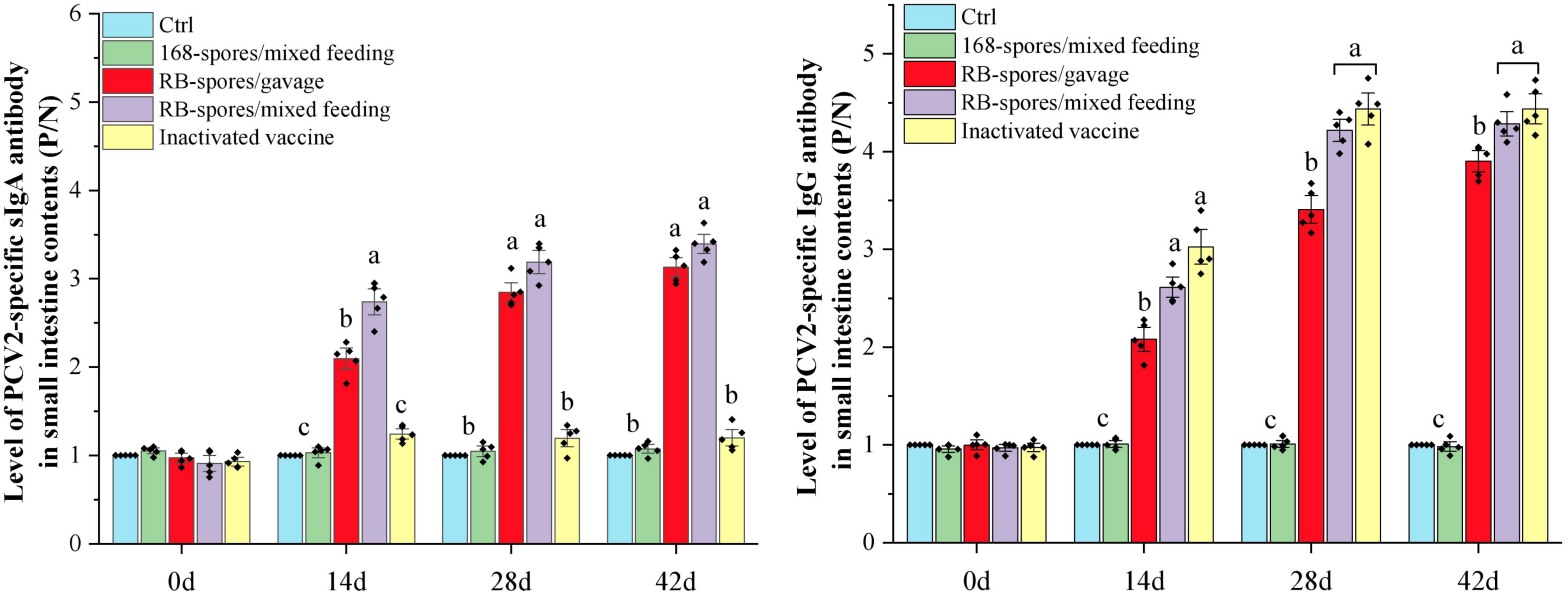
Immune responses after immunizing mice with different methods. The levels of sIgA in the small intestinal contents and IgG levels in serum of experimental mice at 0d, 14d, 28d, and 42d were expressed as *P/N* values. Data were presented as mean ± standard deviation (*n*=5). One-way ANOVA followed by a Friedman test was performed, bars with different letters indicate significant difference (*P* < 0.05).

Mucosal and systemic immune responses of specific pathogen free (SPF) BALB/c mice fed with spores of *B. subtilis* RB were further analyzed (**Figure 4**). Only orally immunized with recombinant spores could induce high level of sIgA in small intestinal mucosa. The sIgA in RB-spores/mixed feeding group reached the highest level on day 28 and stabilized. In RB-spores/gavage group, with the increase of gavage times, sIgA in small intestinal contents and IgG in serum gradually increased. sIgA in small intestinal contents of RB-spores/gavage group was significantly lower than that in RB-spores/mixed feeding group at day 14 (*P*<0.05). From day 28 to 42, although it was still lower than that in RB-spores/mixed feeding group, the difference was not statistically significant (*P*≥0.05). In contrast, intraperitoneal injection of commercial inactivated vaccines could not induce effective intestinal mucosal immunity in mice. But it induced the highest levels of serum IgG in all experimental groups throughout the experimental period. Although the serum IgG contents of RB-spores/mixed feeding group was always lower than that of inactivated vaccine group during the experiment, the difference between them was not significant (*P*≥0.05). In contrast, RB-spores/gavage consistently produced less serum IgG than the RB-spores/mixed feeding group and the inactivated vaccine group during the trial (*P*<0.05).

### Virus microneutralization test

**Figure 5:**
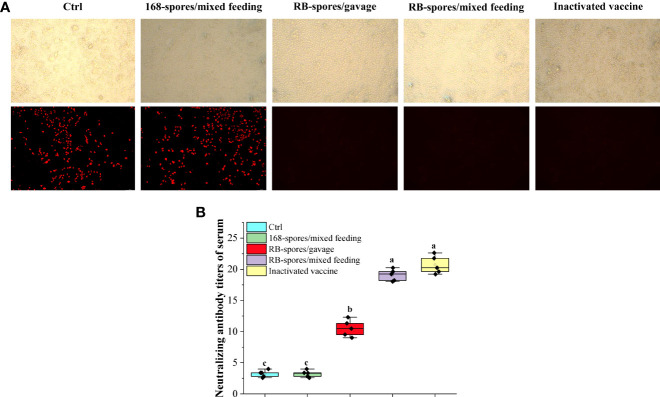
Virus microneutralization test. **(A)** Sera from each group were co-incubated with 50 μL of 200 TCID50 of *PCV2* and then inoculated with pre-cultured PK15 and grown to 72h for indirect immunofluorescence assay. The figures in the upper row represent the morphology of cells in the bright field of fluorescence microscopy. The second row indicates the cells in the fluorescent field of view. (Magnification of 20×) **(B)** Determination of neutralizing antibody titers in serum of each group by Reed-Muench method. Spores displaying heterologous antigens on the surface could induce high titers of serum neutralizing antibodies. Data are presented as means ± standard deviation (n=5). One-way ANOVA followed by a Friedman test was performed, bars with different letters indicate significant difference (*P* < 0.05).

*PCV2* SC2020 was isolated and inoculated into PK15 cells. The TCID50 of *PCV2* SC2020 measured by IPMA method was 10^-5.1^. PK15 cells were pre-cultured in 96-well plates until confluence reached 50-60%, and then virus microneutralization test was carried out. If serum antibodies neutralized *PCV2* virus, it lost the ability to infect cells and has no fluorescent signal in the field (**Figure 5A**). It can be seen that spores of *B. subtilis* 168 failed to stimulate the production of effective neutralizing antibodies (NA) in serum. The neutralization titers of mice immunized with inactivated vaccine and *B. subtilis* RB/mixed feeding were much higher than those of the blank control group (*P* < 0.05). As shown in **Figure 5B**, mice immunized with commercial vaccines triggered NA titers of 1:21.1 on 42nd day and mice in RB/mixed feeding group developed mean NA titers of 1:19.2. The titers of serum NA in RB/gavage only reached 1:10.7, which were significantly lower than that in RB/mixed feeding group (*P*< 0.05).

### Quantitative Real-time PCR detection of cytokine expression in small intestine

**Figure 6:**
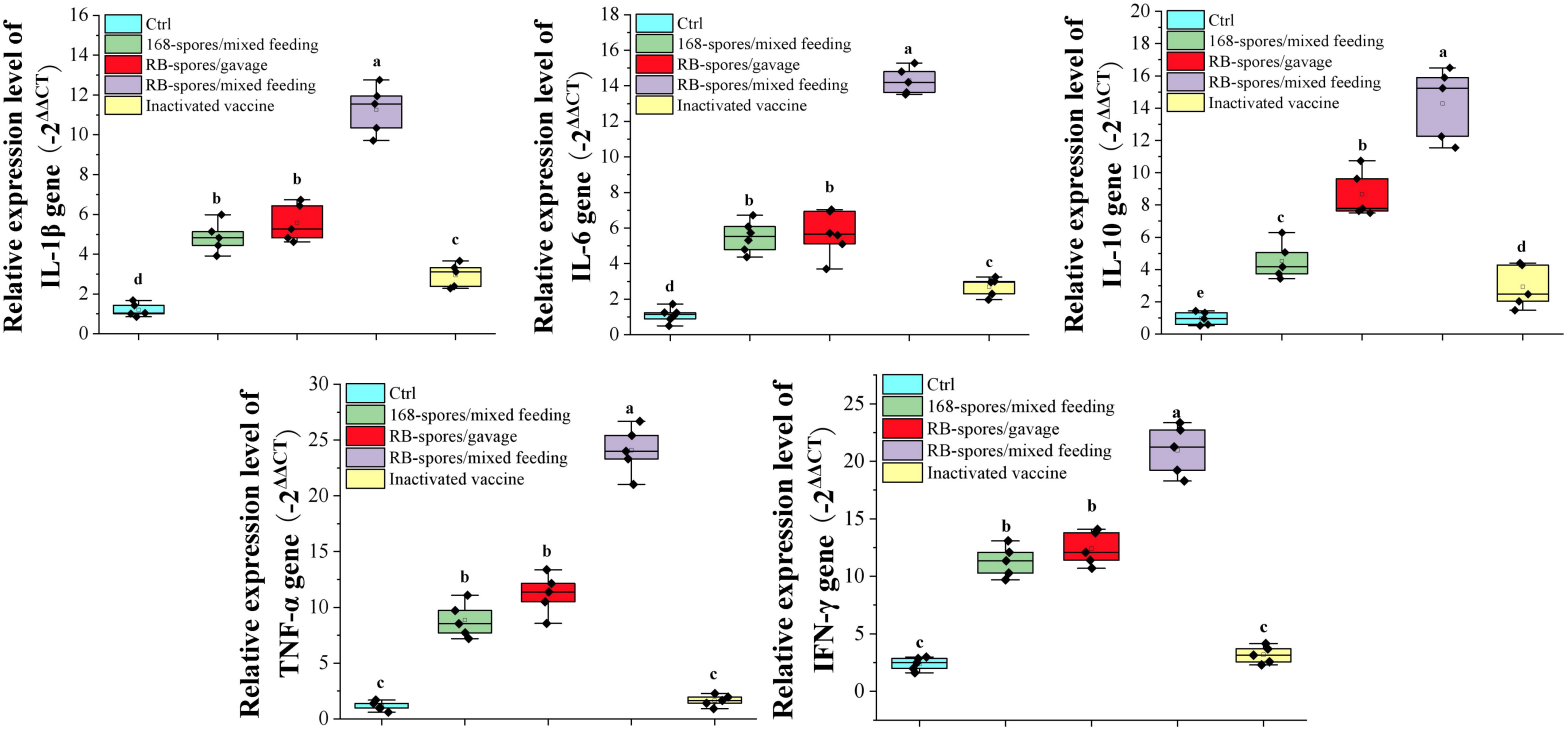
Expression of small intestine cytokines detected by quantitative real-time PCR. The expressio*n* levels of cytokines IL-1β, IL-6, IL-10, TNF-α and IFN-γ were expressed by the value of -2^ΔΔCT^. Data are presented as mean ± standard deviation (n=5). One-way ANOVA followed by a Friedman test was performed, bars with different letters indicate significant difference (*P* < 0.05).

Total RNA was extracted from small intestine tissue of mice on day 42 and performed reverse transcription. Using the obtained cDNA as template, quantitative real-time PCR was performed to determine what kind of immune related genes were upregulated in response to *B. subtilis* spores (**Figure 6**). The expression of IL-1β, IL-6, IL-10, TNF-α and IFN-γ was significantly increased in small intestinal mucosa stimulated with the *B. subtilis* spore (*P*<0.05). It can be seen that the immunoregulation effect of mixed feeding was better than that of gavage (*P*<0.05). In addition, immunomodulatory effect of recombinant spores was significantly better than that of wild-type spores (*P*<0.05). These results indicated that the *B. subtilis’* spores had good mucosal immunoadjuvant function, and mix feeding is a more appropriate method of giving spores. After intraperitoneal injection of inactivated vaccine, the expression of IL-1β, IL-6 and IL-10in ileum tissue was significantly increased compared with the blank control group (*P*<0.05). However, its expression level was much lower than that of spore-immunized group (*P*<0.05). Apparently, the inactivated vaccine upregulated the expression of cytokines in the intestinal mucosa by regulating the systemic immune response, which was far less effective than the direct effect of spores at the mucosa.

### Detection of CD3+, CD4+, CD8+ T lymphocytes and IELs in small intestine

**Figure 7:**
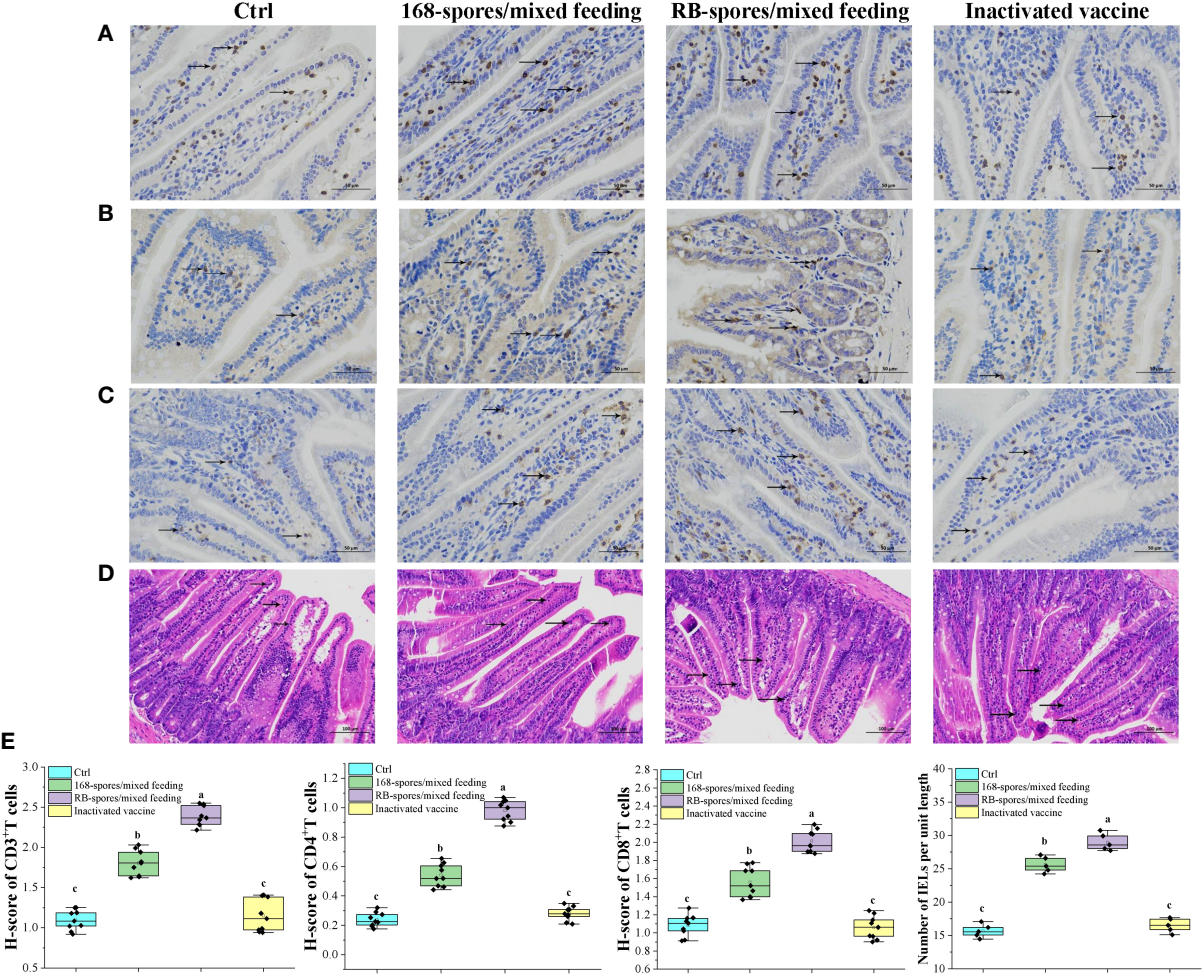
The distribution of CD3+, CD4+, CD8+ T lymphocytes and IELs in intestinal mucosa was observed and quantitatively analyzed. **(A–C)** Distribution of CD3+, CD4+ and CD8+ T lymphocytes in the ileal mucosa, black arrows indicate typical positive cells. Magnification of 400×. (n=9) **(D)** Distribution of IELs cells in the small intestinal mucosa. IELs are shown by black arrows, which are distributed in the mucosal epithelial layer of the small intestine, with irregular nuclei and darker staining than epithelium. Magnification of 200×. (n=5) **(E)** The number of CD3+, CD4+ and CD8+ T lymphocytes in the intestinal mucosa, expressed as H-score. And the number of IELs per unit length in the intestinal villi. H-score is a value between 0-300, the larger the value, the stronger the comprehensive positive intensity and the more positive cells. Data are presented as means ± standard deviation. One-way ANOVA followed by a Friedman test was performed, bars with different letters indicate a significant difference (*P* < 0.05).

Immunohistochemical analysis was performed on small intestinal tissue slices after antigen retrieval. After DAB substrate treatment, the nuclei of positive cells were light yellow (weak positivity) or tan (positive), and the micrographs showed representative CD3+, CD4+, CD8+ T lymphocytes and IELs (**Figures 7A–D**, as indicated by the black arrow). The results showed that the comprehensive positive intensity of CD3+ and CD4+ T lymphocytes in the small intestinal mucosa of 168/mixed feeding group and RB/mixed feeding group were significantly higher than that of blank control group and vaccine group (*P*<0.05). The H-score of CD3+ and CD4+ T lymphocytes in RB/mixed feeding group was significantly higher than that in 168/mixed feeding group (*P*<0.05) (**Figure 7E**). In addition, the positive intensity of CD8+ T lymphocytes in 168/mixed feeding group and RB/mixed feeding group was significantly higher than that in blank control group and vaccine group (*P*<0.05). These results suggested that both *B. subtilis* RB and 168 spores can promote the differentiation of intestinal mucosal immune lymphocytes, and the spores of *B. subtilis* RB had a better effect.

The number of IELs per unit length of intestinal villi was significantly increased in spore-immunized group compared with blank control group and vaccination group (*P*<0.05). In all data, there was no significant difference between blank control and vaccination group (*P*≥0.05). These results indicated that spores can effectively promote the immune response of intestinal mucosa as well as the proliferation and differentiation of lymphocytes. In addition, the promotion effect of *B. subtilis* RB was better than that of *B. subtilis* 168. Intraperitoneal injection of inactivated vaccine had no such effect.

## Discussion

The main reason for the widespread prevalence and rapid evolution of *PCV2* is that it mainly infects animals through respiratory tract and gastrointestinal mucosa. Infection with *PCV2* caused necrosis of ileal mucosal cells and inhibition of mucosal coagulation in piglets. In addition, it altered the expression of immune-related genes such as CCL4, CCL5 and CCL28, induced accelerated apoptosis of intestinal mucosal lymphocytes, altered immune cell subsets and inhibited production of ileal sIgA (37). Damage to the intestinal mucosa - the first defense barrier of the animal’s intestine - in turn leads to more pathogens that can easily pass through the intestinal mucosa to attack the animal. However, current inactivated vaccines and *Cap* protein-based subunit vaccines cannot induce effective mucosal immunity after intramuscular injection (37, 38). The coat protein of spores is considered an ideal platform for antigen delivery because of its excellent mucosal immune adjuvant properties and strong resistance to harsh physical and chemical environments. *Cap* protein-based *PCV2* subunit vaccines have been reported in many studies (39–41). In addition, the 47 amino acids at the N-terminal of *Cap* protein were NLS (nuclear localization signal) peptides, which did not contain major antigenic epitopes (42). However, the presence of NLS may result in the expressed *Cap* protein being transported to the nucleus and expressed as an insoluble protein (43), which is not favorable for display on the spore surface. In this study, truncated *Cap* protein was successfully displayed on the spore surface of *B. subtilis*, with 47 amino acids removed from the N-terminal.

In this study, a 579 bp truncated *Cap* gene was amplified from spleen homogenates of *PCV2*-infected pigs with primers. After preliminary examination, there was no mixed infection with other viruses in tissue homogenate. In addition, the truncated *Cap* gene of this strain was 100% homologous with *PCV2* CH/HNZMD1/201406 (GenBank Accession: KX641112.1), which belonged to *PCV2*d and was named *PCV2* SC2020. *PCV2*d is the most prevalent genotype worldwide (6, 44, 45). The use of commercial or experimental vaccines based on different genotypes can also provide immune protection against other *PCV2* genotypes in animals. However, in the context of the current *PCV2* epidemic, it is more targeted to use the *PCV2d* gene as a vaccine candidate (7, 46). In this study, *tCap* protein was successfully expressed in prokaryotes with the correct size and responsiveness to *PCV2* polyclonal antibodies. The *tCap* and *cotB* genes constituted a fusion gene and were inserted into the genome of *B. subtilis* 168 by pDG364. After verification, a recombinant *B. subtilis* RB was successfully constructed, and *tCap* could be correctly displayed on the spore surface.

Effective mucosal vaccines not only provide mucosal immunity, but also have the ability to induce systemic immune protection. Although *Bacillus subtilis* is a resident soil organism, spores cannot be considered simply as a food, rather, they generate specific local and systemic immune responses. But Permpoonpattana et al. found the immune response they induced was non-specific, indicative signs of the stimulation of innate immunity (47). Microfold (M cells) cells are responsible for the uptake and presentation of antigens to dendritic cells (DCs) in mucosa-associated lymphoid tissue to initiate an immune response (48, 49). Duc et al. found that not only humoral immunity but also a cellular immune response may be generated after spores mixed feeding to mice. This is because the IgG2a subclass predominates over IgG1 in the early stages of immunity (50). Compelling evidence suggests that the dominance of this subclass indicates a type 1 (Th1) T cell response leading to CTL recruitment and IgG synthesis, an increase in IgG2b in the late phase of immunization suggests a type 2 (Th2) T-cell response and explains the sIgA/IgG1 response (51, 52). Limited survival of the vegetative B. subtilis cell in macrophages has been demonstrated previously (53). Although the vegetative cell is ultimately destroyed, the production of specific sIgA and IgG proved that there was still sufficient time for the *Cap* proteins on the surface of the recombinant spores to be recognized to induce a specific immune response. We believe that it is this mechanism of germination and destruction within phagocytes that makes *Bacillus subtilis* immunostimulatory and non-invasive as a harmless probiotic. In this study, *B. subtilis* RB induced high levels of serum IgG and intestinal sIgA. In addition, the immunization effect of mixed feeding was better than that of limited frequency of gavage. Leser (54) showed that because *B. subtilis* was an aerobic bacterium, most probiotic spores added to diets will germinate in the proximal part of the digestive tract, and the vegetative growth will only be very limited. In addition, it has been shown that *Bacillus subtilis* can grow in an anaerobic environment with limited (55, 56). They could remain in the gastrointestinal tract temporarily rather than permanently colonizing. The experimental animals in RB-spores/gavage group were only given a limited number of gavages, which may cause the spores unable to effectively linger on the mucosa to induce immunity and then be excreted from the body. While the addition of *B. subtilis* RB’s spores in the diet could stimulate the intestinal mucosa continually. No effective *PCV2*-specific sIgA was detected in the intestinal contents of experimental animals immunized with inactivated vaccine. Because most commercial vaccines administered *via* the parenteral route fail to induce protective mucosal immunity, they may only provide protection against clinical disease and do not eliminate infection at the site of local mucosal invasion (17). In addition, at 42nd, the serum IgG contents of RB-spores/mixed feeding group was still lower than that of the inactivated vaccine group, but the difference between them was not significant. Similar results were obtained by Seo et al. who compared the immunization effects of several inactivated and subunit vaccines, inactivated *PCV2* vaccines induced higher levels of *PCV2*-specific NA in pigs compared to subunit vaccines (57). The immune adjuvants used in our commercial inactivated vaccines was conventional aluminum adjuvants, it may promote antibody formation at the expense of cell-mediated immunity (58–60). Oleszycka et al. found that injection of aluminum adjuvant inhibited Th1-like responses by promoting increased secretion of serum IL-10 in mice (61). IL-10-deficient mice are less susceptible to Mycobacterium tuberculosis infection which is accompanied by early Th1 responses in the lung (62). Moreover, blockade of IL-10 signaling during BCG (Bacillus Calmette-Guérin) vaccination enhances Th1 and Th17 responses and increases protection to Mycobacterium tuberculosis infection (63). It has also been shown previously that Th1 responses induced by Complete Freund’s Adjuvant are suppressed by IL-10, as IL-10-deficient mice secrete elevated antigen-specific IFN-γ when lymph node cells are restimulated ex vivo (61). This conclusion was confirmed by our subsequent trials. It was seen that in the inactivated vaccine immunized group, the expression level of IL-10 at the small intestinal mucosa was significantly increased, while TNF-α and IFN-γ, important regulator of cellular immunity, did not show an upregulation trend. However, it has been demonstrated that the onset of T-cell immune response occurs in parallel with the reduction in *PCV2* viremia, while neutralizing antibodies do not appear until several days later (64), and therefore cell-mediated immunity is necessary for eventual viral clearance. Besides we believed this may also be due to the lack of a peptide linker between the *cotB* protein and the *tCap* protein, which was a limitation of our study. The lack of a peptide linker often caused steric hindrance between the two fusion proteins, resulting in low efficiency in displaying *tCap (* 65). Negri compared *cotB*-*FliD* and *cotB*-linker-*FliD* two ways of displaying *C. difficile FliD* antigen protein. They found that the former only displayed 6.9×10^2^ antigenic proteins on the surface of each spore, while the latter displayed 1.1×10^3^ proteins (66). Potocki et al. (21)demonstrated that more efficient display of antigenic proteins on the surface of the budding spore can effectively increase the immunogenicity of the recombinant spore, thereby inducing a stronger immune response. Improving the display efficiency of *tCap* protein on the surface of *B. subtilis* 168 by adding different linker peptides may be the main content of our next work.

Furthermore, we constructed an *in vitro* infection model of *PCV2* using porcine kidney epithelial-like cells PK15. Virus neutralization test confirmed that mice immunized with *B. subtilis* RB could induce effective serum neutralizing antibodies (NA). The average titer of NA in RB-spores/mixed feeding group was 1:19.2, while it only reached 1:10.7 in RB-spores/gavage group. In Chen’s (25)study, they used a polycistronic *baculovirus* to display 4*tCap* proteins on its surface. They found that the serum neutralizing antibody titers reached 1:11. But in our research, the maximum value was 1:19.2 when mice were mixed feeding with *B. subtilis* RB. The higher titer of neutralizing antibody indicated that *B. subtilis’* spores was a good mucosal immune adjuvant. Lower serum IgG levels and neutralizing antibody titers at 42d suggested that gavage feeding of recombinant spores was not an appropriate approach. Interestingly, even when recombinant spores are immunized in an appropriate manner (mixed feeding), the potency of the induced serum IgG and serum neutralizing antibodies does not exceed that of inactivated vaccines. Opriessnig et al. (67)came up with results similar to ours. They used a baculovirus-vectored subunit vaccine VAC-2 and a whole virus inactivated vaccine VAC-1 to immunize growing pigs. They found that the inactivated vaccine induced higher levels of IgG and neutralizing antibodies than the subunit vaccine. However, VAC-2 can more significantly reduce the viral load in serum. There is no significant correlation between antibody levels induced by different vaccines and their ability to reduce viremia. The lack of correlation between antibody levels and *PCV2* viral load may be a result of the subunit vaccine inducing a cellular immune response that is unrelated to the serum antibody response.

The current absence of a consistent, accurate and reproducible PMWS model is one of the main obstacles to establish clear parameters for the experimental evaluation of *PCV2* vaccine (68). The prevailing parameters for assessing vaccine protective capacity based on PMWS diagnostic criteria at this stage include (1) the presence of clinical signs; (2) the presence of characteristic pathological changes under microscopy; (3) the detection of *PCV2* at the site of the lesion; and (4) the viral load in the serum associated with clinical signs. Although we demonstrated that the spores of *B. subtilis* RB can effectively induce mucosal and systemic immune responses in experimental mice, it still requires more detailed evaluation of the protective capacity in the natural host, which will be another focus of our next work.

Inflammatory cytokines are key regulators of the animal immune system in regulating innate and adaptive immune responses (69). IFN-γ is the main effector of cellular immunity, which can activate CD8+ T lymphocytes and macrophages to kill foreign pathogens or infected cells (25). Most commercial vaccines focused only on humoral responses, including antigen-specific or neutralizing antibody responses; however, failed to induce strong cellular immunity. This is because the presentation of heterologous antigens is biased towards MHC II molecules to promote Th2-type response rather than CD8+ T cell-mediated cytotoxic T lymphocyte (CTL) response (70). Studies have found that spores of *B. subtilis* can germinate into vegetative cells after being taken up by DC cells, resulting in the presence of antigens in DC cells (50). DC cells are unlikely to provide a nutritionally attractive environment, but rather phagosomes/phagolysosomes may somehow chemically mimic the conditions required for spore germination, thereby facilitating germination and rapid destruction of bacterial cells (50). This promotes antigen presentation towards MHC I, which induces CTL response. CD3 + T lymphocytes are an important class of lymphocytes that play a key role in the activation of CD8 + T and CD4 + T lymphocytes. Intraepithelial lymphocytes (IELs), existed in the epithelial layer of mucosal tissues, are involved in the recognition of and defense against pathogens (71). We found that the spores of *B. subtilis* RB could effectively promote the proliferation of IELs, while the inactivated vaccine could not. Quantitative analysis by H-scores method showed that it also promoted the proliferation and differentiation of CD3+, CD4+ and CD8+ T lymphocytes, and the effect of mixed feeding was significantly better than that of gavage. In Zhang’s (38) research, the proliferation of CD3+ T and CD4+ T cells in the small intestinal mucosa of experimental animals both increased under the influence of recombinant *B. subtilis* WB800N. However, the proliferation of CD8+ T cells was not affected. We believed the main reason for this was that they did not spore the recombinant *B. subtilis* WB800N before feeding the animals. This resulted in a large number of *B. subtilis* failing to sporulate, and cannot play the function of balancing Th1/Th2 immune responses (72). *B. subtilis* RB significantly up-regulated the expression level of IL-1β, IL-6, IL-10, TNF-α and IFN-γ gene. Among them, IL-1β, TNF-α and IFN-γ indicated the polarizations of Th1-immune response while IL-6 and IL-10 indicated the polarizations of Th2 (72). In addition, the spores of *B. subtilis* 168 also had an up-regulation effect. These soluble cytokines enhance the proliferation of T and B cells and subsequently enhance antigen-specific adaptive immune responses. This proved that spore itself had excellent immune adjuvant function and could balance Th1/Th2 immune response effectively, which was consistent with the conclusion of Lee et al (73). They confirmed that the Spore + inactivated H9N2 virus group could induce a stronger specific immune response than the inactivated H9N2 virus group. These results indicated that the spores of *B. subtilis* as a feed additive can significantly promote the proliferation of immune-related lymphocytes in animals, and is a good mucosal immunoadjuvant. Although we have shown that B. subtilis spores increased the proliferation of CD8+ and CD4+ T cells, we need more sophisticated *in vitro* and *in vivo* models to evaluate the molecular mechanism of cross-presentation of foreign antigens in antigen-presenting cells.

In conclusion, we successfully constructed a recombinant *B. subtilis* that displayed *PCV2 tCap* protein on the spore surface. The vaccine is a novel oral mucosal vaccine that has shown potent immune properties in mouse models and could induce high levels of antibodies in the mucosal and systemic systems. Serum antibody can effectively neutralize *PCV2*. Spores of *B. subtilis* RB not only up-regulated the expression level of intestinal inflammatory cytokines, but also effectively balanced the Th1/Th2 immune response. In addition, the recombination spores can effectively promote the proliferation of related lymphocytes. These results indicate that recombinant *B. subtilis* RB is a promising candidate for the development of new *PCV2* vaccines.

## Data availability statement

The datasets presented in this study can be found in online repositories. The names of the repository/repositories and accession number(s) can be found in the article/**Supplementary Material**.

## Ethics statement

The animal study was reviewed and approved by Institutional Animal Care and Use Committee of Sichuan Agricultural University (approval number: DY2020203062).

## Author contributions

WL, JL, and XD contributed equally to this study. WL, JL, and KP designed the research. WL, JL, XD, and ML performed the experiments and analyzed the data. ZW performed the statistical analyses. DMZ and BJ contributed reagents and materials. YZ, XN, and DZ performed review and editing. AK checked the language proficiency. WL and KP organized and drafted the paper. All authors contributed to the article and approved the submitted version.

## Funding

The present study was supported by the Technology Innovation Research Team in the University of Sichuan Province (Award Number: KM406183.1), and Sichuan Agricultural University Double Branch Program (Award Number: 03571146).

## Conflict of interest

The authors declare that the research was conducted in the absence of any commercial or financial relationships that could be construed as a potential conflict of interest.

## Publisher’s note

All claims expressed in this article are solely those of the authors and do not necessarily represent those of their affiliated organizations, or those of the publisher, the editors and the reviewers. Any product that may be evaluated in this article, or claim that may be made by its manufacturer, is not guaranteed or endorsed by the publisher.
