# Supplementary material for: Surface Display of porcine circovirus type 2 antigen protein cap on the spores of bacillus subtilis 168: An effective mucosal vaccine candidate
Source: Front Immunol. 2022 Sep 15;13:1007202. doi: 10.3389/fimmu.2022.1007202 (PMC9520567; doi:10.3389/fimmu.2022.1007202)
Supplement: Supplementary file 1 [file DataSheet_1.pdf]

Supplementary materials for

Surface Display of Porcine Circovirus Type 2 Antigen Protein *Cap* on the Spores of *Bacillus subtilis 168*: An effective mucosal vaccine candidate

Weijie Li [1], Jianzhen Li [1,2], Xixi Dai [3], Minggang Liu [1], Abdul Khalique [1], Zhenghua Wang [2], Yan Zeng [1], Dongmei Zhang [1], Xueqin Ni [1], Dong Zeng [1], Bo Jing [1], Kangcheng Pan [1*]

[1]Animal Microecology Institute, Department of Animal and Plant Quarantine, College of Veterinary Medicine, Sichuan Agricultural University, Chengdu 611100, China

[2]College of Animal Husbandry and Veterinary, Chengdu Agricultural College, Chengdu 611100, China;

[3]College of Animal Science and Technology, Chongqing Three Gorges Vocational College, Chongqing 404100, China

*** Correspondence:**

Corresponding Author**:** +86 28 86290111; Fax: +86 28 82652669;

E-mail address: pankangcheng71@126.com

The file includes:

Supplementary Tab. S1 The bacteria, virus strains, cell lines and plasmids used in this study.

Supplementary Tab. S2 Representative strains of different *PCV2* genotypes used to construct phylogenetic tree.

Supplementary Tab. S3 The primer sequences used for qPCR.

Supplementary Tab. S4 tCap gene sequences and optimized sites.

Supplementary Fig. S1 Cloning of truncated *Cap* gene and virus typing.

Supplementary Fig. S2 Validation of recombinant integration plasmid pDG364-*cotB*-*tCap2*.

Table S1 Strains, Cell, Plasmids and primers sequences.

Plasmids, Strains, and Primers Sequences	Description	Source or Restriction Site
**Strains**		
Porcine circovirus type 2	Spleen carrying the virus were collected from an infected pig at a pig farm in Sichuan in 2020, strain was named *PCV2* SC2020	From Pig Farm Health Testing and Evaluation Center of Sichuan Agricultural University
E. coli DH5α and BL21	Type strain	TIANGEN Biotech (Beijing)
B. subtilis 168	GenBank Accession: AL009126.3	Our lab
B. subtilis RB	B. subtilis 168 *amyE:: cotB-tCap*	This work
**Cell**		
PK15 cell line	Type strain	Our lab
**Plasmids**		
pUCm-T	T-A Cloning vector	Sangon Biotech (Shanghai)
pET32a-*tCap*	prokaryotic expression vector	This work
pDG364	*E. coli-B. subtilis* shuttle vector	Our lab
pDG364-*cotB*	pDG364 derivative carrying *cotB* gene	Our previous work(17)
pDG364*cotB-tCap*	pDG364 derivative carrying the fusion *cotB-tCap* gene	This work
**Primer sequences**		
tCap-F1	5'- CCGGGATCC*ATGAATGGCATCTTC -3'	*BamH I*
tCap-R1	5'-CGCGAATTCTTAGGGTTTAAGTGGGGGGTC-3'	*EcoR I*
tCap-F2	5'-CCGAAGCTTATGAATGGCATCTTC -3'	*Hind III*
tCap-R2	5'-CGCGAATTCTTAGGGTTTAAGTGGGGGGTC-3'	*EcoR I*
cotB-F	5'-CGGGATCCACGGATTAGGCCGTTTGTCC-3'	*BamH I*
cotB-R	5'-GGGAAGCTTGGATGATTGATCATCTGAAG-3'	*Hind III*
amyE-F	5'-CCAATGAGGTTAAGAGTATTCC-3'	Null
amyE-R	5'-CGAGAAGCTATCACCGCCCAGC-3'	Null

Table S2 Different strains of *PCV2* used to construct the phylogenetic tree

Genotype	Strains	Accession number
PCV2a	*Porcine circovirus 2* strain Canada	AF055392.1
	Porcine circovirus 2 strain CL	HM038033.1
	Porcine circovirus 2 strain LG	HM038034.1
PCV2b	*Porcine circovirus 2* strain TZ0601	EU257511.1
	Porcine circovirus 2 strain YJ,	HM038032.1
	Porcine circovirus 2 strain am5	DQ856567.1
	Porcine circovirus 2 strain 05-55004-7	HQ713495.1
	Porcine circovirus 2 from France	AF055394.1
	Porcine circovirus 2 strain BJ0401	EF524515.1
PCV2d	Porcine circovirus type 2 strain TJ	AY181946.1
	Porcine circovirus 2 strain CH/HNZMD1/201406	KX641112.1
	Porcine circovirus 2 strain GDYX	JX519293.1
	Porcine circovirus 2 strain AH	HM038030.1
	Porcine circovirus 2 strain BDH	HM038017.1
	Porcine circovirus 2 strain CH/HBWH3/201310	KX641085.1
PCV2c	*Porcine circovirus 2* DK1990PMWSfree	EU148505.1
	Porcine circovirus 2 DK1987PMWSfree	EU148504.1
	Porcine circovirus 2 isolate DK1980PMWSfree	EU148503.1

Table S3 Primers' sequence of quantitative PCR.

Gene	Accession number	Primer sequences	
β-actin	NM_007393.5	F: GCTCTTTTCCAGCCTTCCTT	R: GATGTCAACGTCACACTT
IL-1β	NM_008361.3	F: ATGAAAGACGGCACCCCAC	R: GCTTGTGCTCTGCTTGTGAG
IL-6	NM_031168.1	F: TGCAAGAGACTTCCATCCAGT	R: GTGAAGTAGGGAAGGCCG
IL-10	NM_010548.2	F: GGTTGCCAAGCCTTATCGGA	R: ACCTGCTCCACTGCCTTGCT
IFN-γ	NM_008337.4	F: TCAAGTGGCATAGATGTGGAAGAA	R: TGGCTCTGCAGGATTTTCATG
TNF-α	NM_001278601.1	F: ACGGCATGGATCTCAAAGAC	R: AGATAGCAAATCGGCTGACG

* The bacterial concentration was adjusted to 2.0×10^{10}CFU/ mL with normal saline. On day 1-3, 14-16, and 28-30, each mouse was given 0.1 mL intragastric administration every day for 3 consecutive days.

Table S4 tCap gene sequences and optimized sites

Gene	Sequences	
	*aagcttatgaatggcatcttcaacacccgcctctcccgcaccatcggttatactgtcaag	60
	aaaaccacagtcagaacgccctcctggaatgtggacatgatgagatttaatattaatgat	120
	tttcttcccccaggaggggggctcaaaccccctcactgtgccctttgaatactacagaata	180
	aggaaggttaaggttgaattctggccctgctccccaatcacccagggtgacaggggagtg	240
tCap	ggctccactgctgttattctagatgataactttgtaacaaaggccaatgccctaacctat	300
	gacccctatgtaaactactcctcccgccataccataacccagcccttctcctaccactcc	360
	cggtactttaccccgaaacctgtccttgataggacaatcgattacttccaacccaataac	420
	aaaagaaatcaactctggctgagactacaaactactggaaatgtagaccatgtaggcctc	480
	ggcactgcgttcgaaaacagtatatacgaccaggactacaatatccgtataaccatgtat	540
	gtacaattcagagaatttaatcttaaagacccccccacttaaaccctaagaattc	594
	aagcttatgaatggcatcttcaacacccgcctctcccgcaccatcggttatactgtcaag	60
	aaaaccacagtcagaacgccctcctggaatgtggacatgatgagatttaatattaatgat	120
	tttcttcccccaggaggggggctcaaaccccctcactgtgccctttgaatactacagaata	180
	aggaaggttaaggttgagttttggccctgctccccaatcacccagggtgacaggggagtg	240
Optimized tCap	ggctccactgctgttattctagatgataactttgtaacaaaggccaatgccctaacctat	300
	gacccctatgtaaactactcctcccgccataccataacccagcccttctcctaccactcc	360
	cggtactttaccccgaaacctgtccttgataggacaatcgattacttccaacccaataac	420
	aaaagaaatcaactctggctgagactacaaactactggaaatgtagaccatgtaggcctc	480
	ggcactgcgttcgaaaacagtatatacgaccaggactacaatatccgtataaccatgtat	540
	gtacaattcagagaatttaatcttaaagacccccccacttaaaccctaagaattc	594

*aagctt is the Hind III restriction site，gaattc is the EcoR I restriction site; the yellow highlighted sequences are the optimized sites; the red highlighted sequences are the ATG promoter.

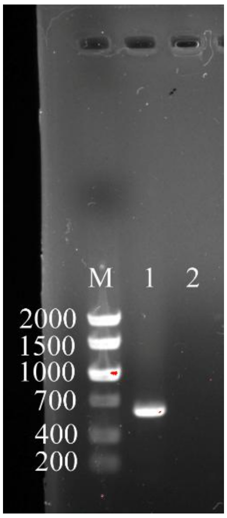

(A)

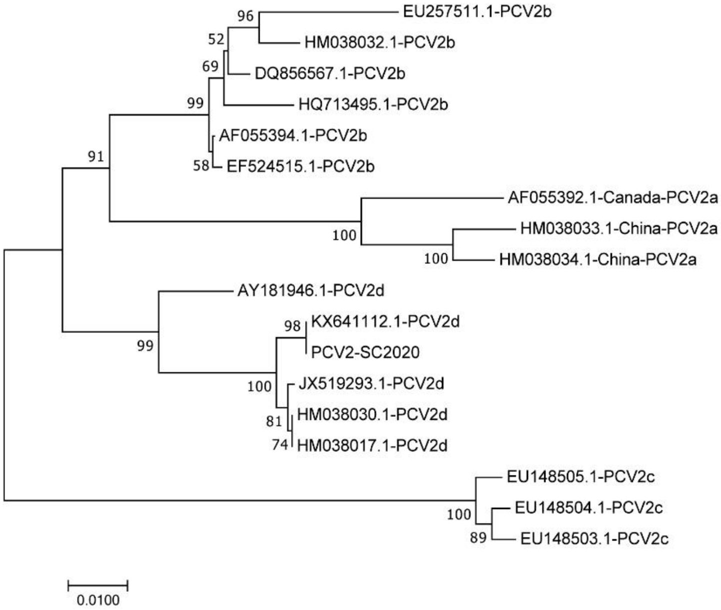

(B)

Fig. S1 Cloning of truncated *Cap* gene and virus typing. **(A)**Agarose gel electrophoresis of target gene. M, 1 and 2 respectively represented DL2000F DNA Marker, PCR product of *tCap* gene and blank control. **(B)**Phylogenetic tree of *PCV2* SC2020 and other reference strains based on *tCap* nucleotide.

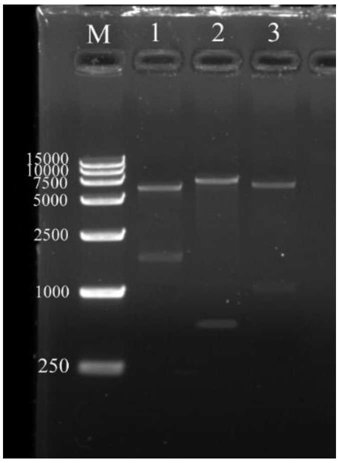

(A)

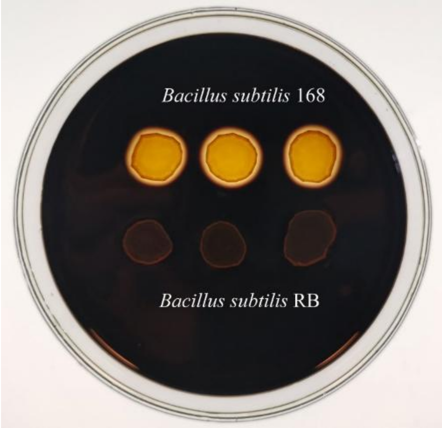

(B)

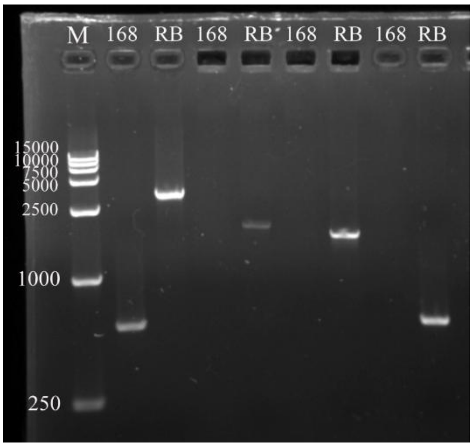

(C)

Fig. S2 Validation of recombinant integration plasmid pDG364-*cotB*-*tCap2*. **(A)** Electrophoretic map of plasmid pDG364-*cotB*-*tCap2* after double digesting with *BamH* I+*EcoR* I, *Hind* III+*EcoR* I, and *BamH* I+*Hind* III, respectively. M, protein marker (250-15000bp); Line 1, *BamH* I+*EcoR* I; Line 2, *Hind* III+ *EcoR* I; Line 3, *BamH* I+*Hind* III. **(B)** *B. subtilis* 168 and *B. subtilis* RB were grown on LB medium containing 1.5% starch for 24 hours and then stained with iodine. **(C)** Agar gel electrophoresis of *B. subtilis* 168 and *B. subtilis* RB genomes amplified with primers *amyE*-F/ *amyE*-R, *amyE*-F/*tCap2*-R, *cotB*-F/*tCap2*-R and *tCap2*-F/*tCap2*-R.
